# MIRAGE syndrome is a rare cause of 46,XY DSD born SGA without adrenal insufficiency

**DOI:** 10.1371/journal.pone.0206184

**Published:** 2018-11-07

**Authors:** Hirohito Shima, Mie Hayashi, Takashi Tachibana, Makoto Oshiro, Naoko Amano, Tomohiro Ishii, Hidenori Haruna, Maki Igarashi, Masafumi Kon, Ryuji Fukuzawa, Yukichi Tanaka, Maki Fukami, Tomonobu Hasegawa, Satoshi Narumi

**Affiliations:** 1 Department of Molecular Endocrinology, National Research Institute for Child Health and Development, Tokyo, Japan; 2 Department of Advanced Pediatric Medicine, Tohoku University School of Medicine, Tokyo, Japan; 3 Department of Pediatrics, Keio University School of Medicine, Tokyo, Japan; 4 Department of Neonatology, Japanese Red Cross Nagoya Daiichi Hospital, Nagoya, Japan; 5 Department of Pediatrics, Nagoya University Graduate School of Medicine, Nagoya, Japan; 6 Department of Pediatrics and Adolescent Medicine, Juntendo University School of Medicine, Tokyo, Japan; 7 Department of Pathology, International University of Health and Welfare, Tokyo, Japan; 8 Department of Pathology, Kanagawa Children’s Medical Center, Yokohama City, Japan; Chinese Academy of Medical Sciences and Peking Union Medical College, CHINA

## Abstract

**Background:**

MIRAGE syndrome, a congenital multisystem disorder due to pathogenic *SAMD9* variants, describes a constellation of clinical features including 46,XY disorders of sex development (DSD), small for gestational age (SGA) and adrenal insufficiency (AI). It is poorly understood whether *SAMD9* variants underlie 46,XY DSD patients born SGA (46,XY DSD SGA) without AI. This study aimed to define the frequency and phenotype of *SAMD9* variants in 46,XY DSD SGA without AI.

**Methods:**

Forty-nine Japanese patients with 46,XY DSD SGA (Quigley scale, 2 to 6; gestational age-matched birth weight percentile, <10) without history of AI were enrolled. The single coding exon of *SAMD9* was PCR-amplified and sequenced for each patient. Pathogenicity of an identified variant was verified *in vitro*. Placenta tissues were obtained from the variant-carrying patient, as well as from another previously described patient, and were analyzed histologically.

**Results:**

In one 46,XY DSD SGA patient, a novel heterozygous *SAMD9* variant, p.Phe1017Val, was identified. Pathogenicity of the mutant was experimentally confirmed. In addition to DSD and SGA, the patient had neonatal thrombocytopenia, severe postnatal grow restriction, chronic diarrhea and susceptibility to infection, all features consistent with MIRAGE, leading to premature death at age 14 months. The patient did not have any manifestations or laboratory findings suggesting AI. Placenta tissues of the two variant-carrying patients were characterized by maldevelopment of distal villi without other findings of maternal underperfusion.

**Conclusions:**

MIRAGE syndrome is a rare cause of 46,XY DSD SGA without AI. This study exemplifies that AI is a common feature of MIRAGE syndrome but that the absence of AI should not rule out a diagnosis of the syndrome.

## Introduction

Disorders of sex development (DSD) are congenital conditions in which development of the chromosomal, gonadal or anatomical sex is atypical [1]. DSD patients are grouped according to the karyotypes: 46,XX DSD, 46,XY DSD and sex chromosome DSD. Manifestations of external genitalia in 46,XY DSD are variable ranging from isolated micropenis to complete-female type. Although genetic studies have broadened our understanding on the pathogenesis for 46,XY DSD, the exact cause of each patient remains mostly unspecified in the clinical setting.

Small for gestational age (SGA) is a term used to describe newborns whose weight and/or length is less than expected for gestational age. SGA can be resulted from both intrinsic and environmental causes, particularly in relation to the delivery of oxygen and nutrients through the placenta. Poyrazoglu *et al*. reported that as many as 17.8% of 46,XY DSD patients in the I-DSD Registry are born SGA [2], providing evidence for the association between 46,XY DSD and SGA. The association is at least partly explained by a common inciting factor, placental insufficiency, that results in fetal malnutrition (related to SGA) and chorionic gonadotropin (HCG) deficiency (related to 46,XY DSD) [3]. It is also noteworthy that 46,XY DSD and SGA are simultaneously observed in several rare genetic defects, including IMAGe syndrome due to *CDKN1C* variants [4] and MIRAGE syndrome due to *SAMD9* variants [5].

MIRAGE syndrome is a recently recognized multisystem disorder characterized by six core features: myelodysplasia, infection, restriction of growth, adrenal hypoplasia, genital phenotypes and enteropathy [5]. The cause of the syndrome is germline heterozygous *SAMD9* variants that usually occur *de novo*. The first two studies of MIRAGE syndrome were reported by endocrinology research laboratories of Japan [5] and United Kingdom [6], and as many as 94% of the *SAMD9* variant-carrying patients had adrenal insufficiency (AI) in these studies. All 15 patients with 46,XY karyotype had external genital abnormalities ranging from hypospadias to complete-female type. Female patients with 46,XX karyotype do not show external genital abnormalities, although dysgenesis of ovaries were shown histologically in two patients [5]. Previous molecular genetic investigation on MIRAGE syndrome have chiefly targeted patients with AI [5, 6]. Among the six core MIRAGE features, AI (with or without DSD) is most frequently associated with singe gene disorders [7, 8], although the other features can be nonspecifically observed in premature infants. At present, it remains unclear whether MIRAGE syndrome could be found in patient cohorts of 46,XY DSD without AI. In the present study, we performed screening of *SAMD9* variants in a Japanese patient cohort of 46,XY DSD SGA without AI to define the frequency and phenotypes of *SAMD9* variants in the patient cohort.

## Materials and methods

### Study subjects

This study was approved by the Ethics Committees at National Center for Child Health and Development, and at Keio University School of Medicine. Written informed consent for the molecular study was obtained from the subjects and/or their parents. Japanese patients with 46,XY DSD SGA without AI were enrolled using the following criteria: (i) external genital abnormality with Quigley scale[9] grades 2 to 6/7 (ii) 46,XY karyotype confirmed by G-banding analysis, (iii) gestational age-matched birth weight percentile less than 10, and (iv) no history of suggestive of AI. The genetic samples were collected in two institutions (Keio University School of Medicine and National Center for Child Health and Development) from all over Japan to investigate genetic bases of 46,XY DSD.

### Variant detection

Genomic DNA was extracted from peripheral leukocytes with a standard technique. *SAMD9* has three exons, and all coding sequences are located in exon 3. Approximately 5-kb single coding exon (*i*.*e*., exon 3) of *SAMD9* was PCR-amplified with Herculase II Fusion DNA Polymerase (Agilent, Santa Clara, CA). Sequences of each PCR product was determined with use of Nextera XT DNA Library Prep Kit (Illumina, San Diego, CA) and a MiSeq sequencer (Illumina). BWA 0.7.12 [10] was used for alignment against the human reference genome (NCBI build 37; hg19). Local realignment, quality score recalibration, and variant calling were performed by GATK 3.7–0 [11] with the default settings. The numbering of amino acid residues was based on NP_060124. The presence of called variants was confirmed by Sanger sequencing.

Identified genetic variants were searched in publicly available polymorphism databases including 1000 Genomes Project (http://www.internationalgenome.org/), gnomAD (http://gnomad.broadinstitute.org/) and dbSNP150 (https://www.ncbi.nlm.nih.gov/projects/SNP/). The deleterious effect on protein function was predicted with computational algorithms including PolyPhen-2 (http://genetics.bwh.harvard.edu/pph2/) and SIFT (http://sift.bii.a-star.edu.sg/).

### Functional characterization of Phe1017Val-SAMD9

A HEK293 cell line that expresses N-terminal FLAG-tagged wildtype (WT) SAMD9 protein in the presence of doxycycline has been described previously [12]. A cell line that expresses Phe1017Val-SAMD9 was established in a similar way using a mutated plasmid prepared with a site-directed mutagenesis technique. Before each experiment, inducible stable cells were treated with 1 μg/mL doxycycline (or vehicle) for 48 hours to allow expression of FLAG-tagged SAMD9 (WT or mutant).

For Western blotting, whole cell lysate was extracted with 1% n-dodecyl-β-D-maltoside (Wako Pure Chemical Industries, Osaka, Japan) in TBS supplemented with protease inhibitor cocktail (cOmplete Protease Inhibitor Cocktail; Sigma-Aldrich, St. Louise, MO). The lysates were subjected to 8% SDS-PAGE, which was followed by Western blotting with anti-FLAG M2 antibody (Sigma-Aldrich) as a primary antibody and horse radish peroxidase-conjugated goat anti-mouse antibody (Sigma-Aldrich) as a secondary antibody.

For evaluation of intracellular localization of FLAG-SAMD9, inducible stable cells were fixed with cold methanol for 10 minutes, and were blocked with 5% bovine serum albumin/TBS for 1 hour at room temperature. Immunofluorescence staining was performed with anti-FLAG M2 antibody as a primary antibody and Alexa555-conjugated goat anti-mouse antibody (Thermo Fisher Scientific, Waltham, MA) as a secondary antibody. Cells were observed under a FLUOVIEW FV1000D confocal laser scanning microscope (Olympus, Tokyo, Japan).

For cell growth assays, inducible stable cells were seeded into 96-well plates at about 5% confluence in 100 μL of culture medium. The degree of confluence was quantified every 3 hours for 144 hours using an IncuCyte ZOOM time-lapse microscope and the object counting algorithm (Essen BioScience, Ann Arbor, MI). Growth curve data are representative of three independent experiments.

### Histological analysis of the placenta

Formalin-fixed paraffin-embedded placenta tissue of the patient with the p.Phe1017Val *SAMD9* variant was analyzed for hematoxylin and eosin staining. We also analyzed placenta tissue of a previously reported MIRAGE syndrome patient with p.Arg459Gln *SAMD9* variant (P1.1 in Ref 5). This patient was born at gestational age 27 weeks with birth weight 545 g (-3.7 SD). He had clinically obvious AI. He had microphallus, hypospadias, and undescended small testes consistent with his 46,XY karyotype. He also suffered from thromobocytopenia, recurrent invasive infection. Chronic diarrhea was not applicable since he died at age 3 months.

## Results

### Characteristics of the patients

**Fig 1:**
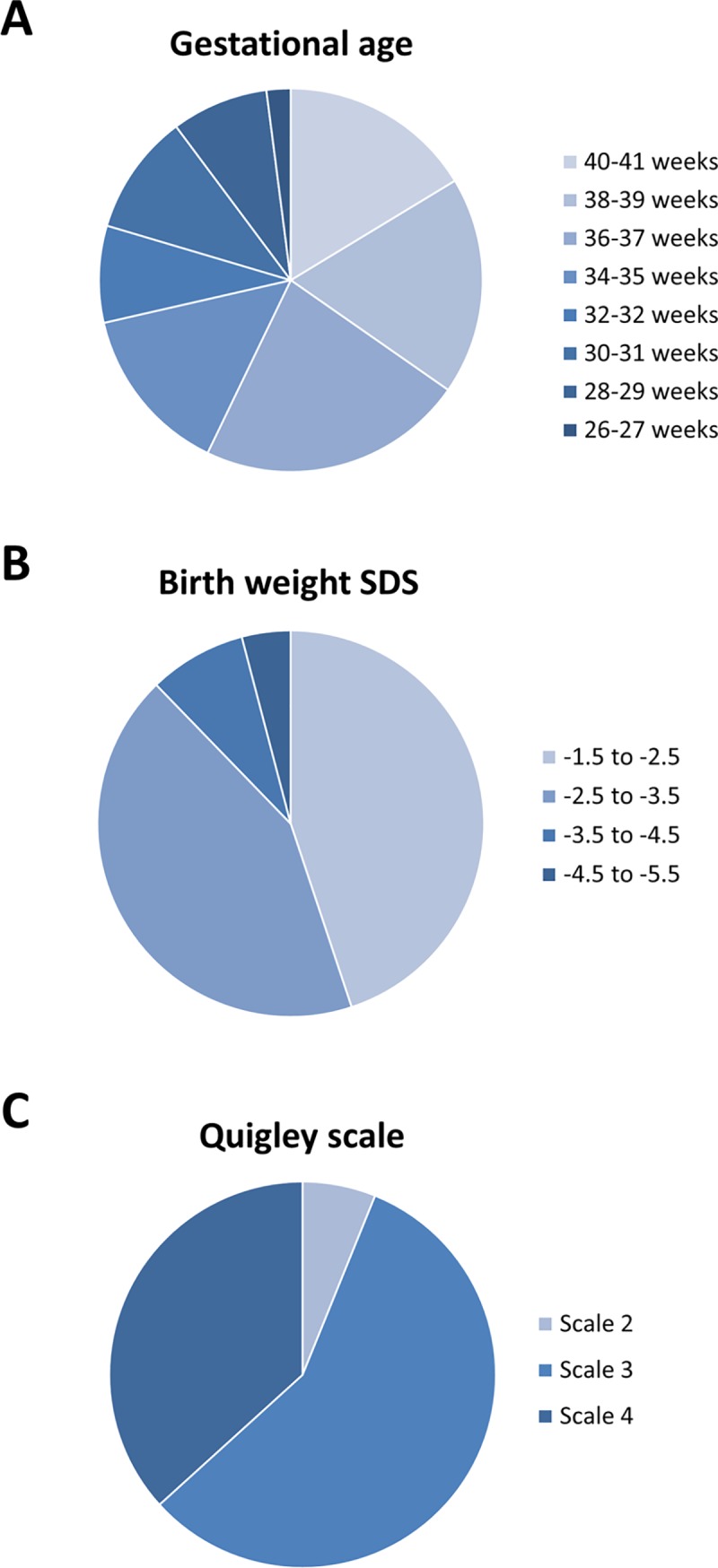
Characteristics of the 49 study subjects are shown, including gestational age (**A**), birth weight SDS (**B**), and disease severity (**C**).

The characteristics of 49 study subjects with 46,XY DSD SGA without AI are summarized in Fig 1. In brief, the median gestational age was 36 weeks, and the median birth weight SDS was -2.4. Quigley scale grades were mostly 3 and 4. All but one subject was reared as male.

### Identification and functional characterization of the Phe1017Val SAMD9 variant

**Fig 2:**
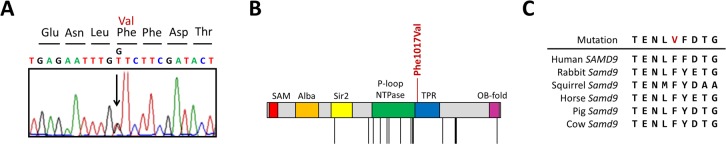
Identification of a novel *SAMD9* variant, p.Phe1017Val. **A,** A partial electropherogram of the PCR product. The patient had a nucleotide substitution of thymine for guanine in the 1017th codon (indicated by an arrow). **B,** A schematic diagram of the SAMD9 protein with domain features. Positions of previously described MIRAGE syndrome-associated variants are indicated by black lines. The p.Phe1017Val variants was located in the TPR repeats. The domain architecture was based on Mekhedov *et al*., *Biol Direct*. 2017;12:13. **C,** Single-letter amino acid Clustal alignments of the residues surrounding the Phe^1017^. The mutated residue is evolutionarily conserved.

The sequence analysis led us to identify one patient with a heterozygous *SAMD9* variant (c.3049T>G, p.Phe1017Val) (Fig 2A). This variant is located in the predicted TPR repeats (Fig 2B), and affects the evolutionarily conserved Phe^1017^ residue (Fig 2C). The variant has not been reported previously, and was absent in any polymorphism databases including 1000 Genomes Project, gnomAD and dbSNP150. The variant was assessed as “possibly damaging” by PolyPhen-2, and as “tolerated” by SIFT. The parents declined parental genotyping.

**Fig 3:**
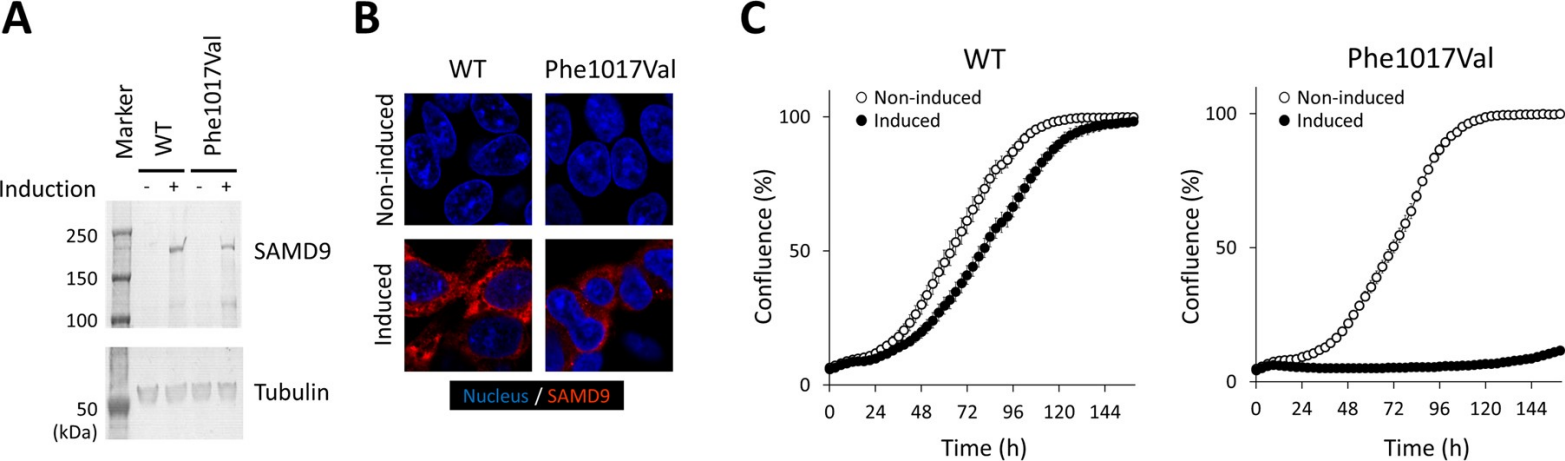
Phe1017Val-SAMD9 was characterized *in vitro* with inducible stable HEK293 cells. **A,** Western blot analysis showed comparable protein expression levels between wildtype (WT)-SAMD9 and Phe1017Val-SAMD9. **B,** Intracellular localization of SAMD9 proteins, evaluated with the immunofluorescence technique, were similar between WT and the mutant. **C,** Induced expression of WT-SAMD9 resulted in mild growth restriction (*left panel*), whereas expression of Phe1017Val-SAMD9 caused profound growth restriction (*right panel*), which is a characteristic of MIRAGE syndrome-associated *SAMD9* variants.

To determine whether Phe1017Val-SAMD9 is a pathogenic variant or a rare benign variant, a series of *in vitro* experiments were conducted. Protein expression levels, assessed by Western blotting, were comparable between WT-SAMD9 and Phe1017Val-SAMD9 (Fig 3A). The immunofluorescent confocal microscopy revealed granular staining pattern in cytoplasm that was similar between WT-SAMD9 and Phe1017Val-SAMD9 (Fig 3B). Assessment of cell growth with use of time-lapse microcopy revealed potent growth-restricting capacity of Phe1017Val-SAMD9 (Fig 3C), which is a common characteristic of MIRAGE syndrome-associated *SAMD9* variants.

### Clinical features of the variant-carrying patient

**Fig 4:**
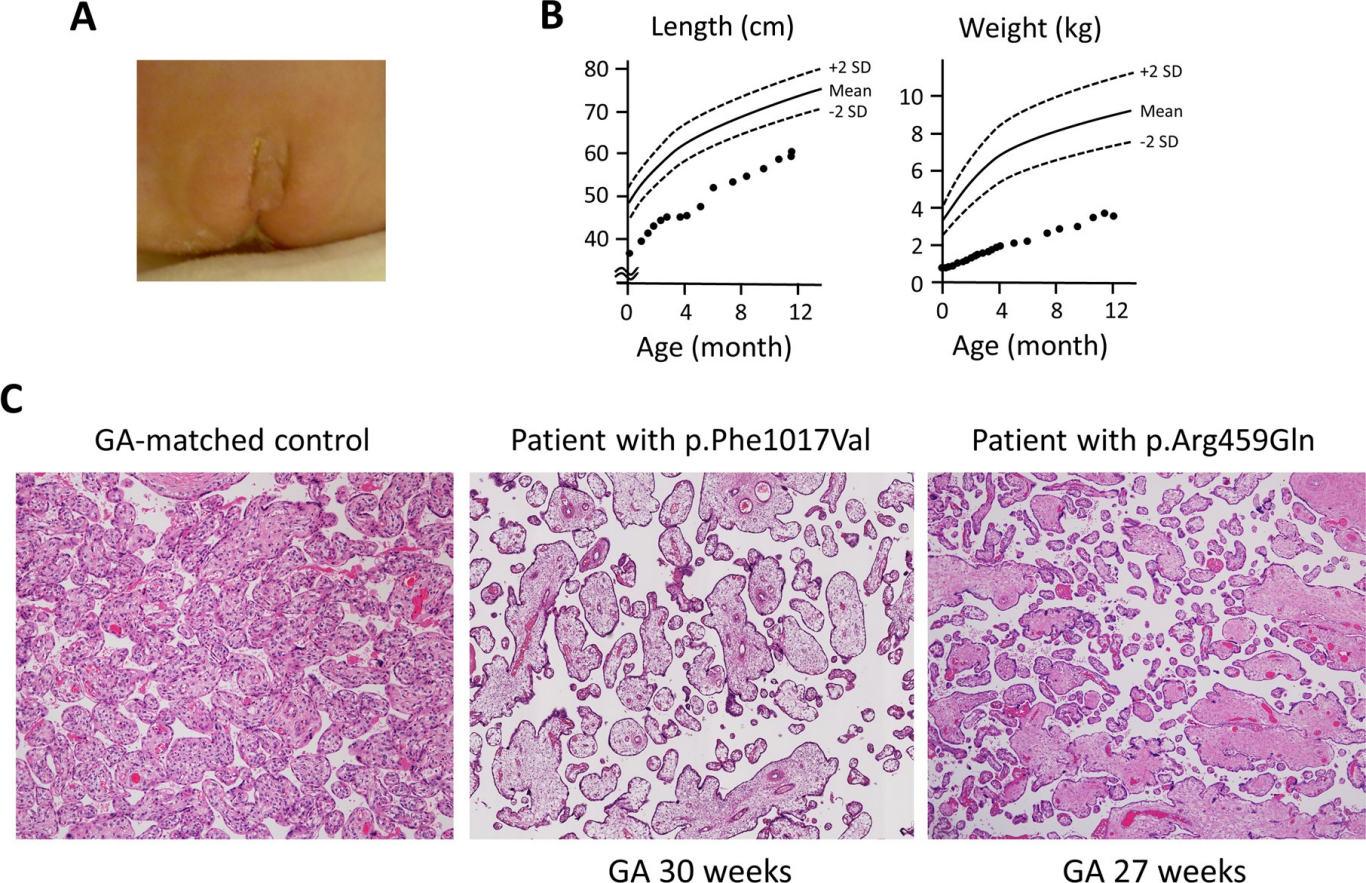
Clinical findings of the patient with the p.Phe1017Val variant. **A,** The patient had significant undervirilization, and the sex of rearing was assigned to female. **B,** Growth restriction was observed in both prenatal and postnatal life (postnatal growth plotted here). **C,** Histological evaluation of the placental tissue derived from two *SAMD9* variant carriers (p.Phe1017Val and p.Arg459Gln) was performed with hematoxylin eosin staining. The two specimens commonly showed sparse, poorly developed distal villous tree with widening of the intervillous space.

The patient with the *SAMD9* p.Phe1017Val variant was born at gestational age 30 weeks by emergency cesarean section due to suspected fetal distress. The parents were healthy and unrelated Japanese individuals. The birth length was 35.6 cm (-1.9 SD), weight 834 g (-2.8 SD), and head circumference 25.6 cm (-1.1 SD). Apgar scores were 2 at 1 min and 6 at 5 min. The weight of the placenta was 220 g (gestational age-matched reference: 208–433 g). The sex of the patient was assigned to female with no specific discussion, though she had mild clitoromegaly (Fig 4A). She developed thrombocytopenia soon after the birth, and received platelet transfusion twice. Neither symptoms nor laboratory findings related to AI, such as skin hyperpigmentation, hemodynamic instability, hypoglycemia or electrolyte abnormalities, were noted throughout the course. From age 1 month, she experienced multiple episodes of gastroesophageal reflux accompanied by aspiration pneumonia, and a duodenal feeding tube was placed. From age 3 months, she had watery loose stool. At age 5 months, she developed hypolacrima with corneal ulcer. The growth was poor (Fig 4B), and she had severe developmental delay: she could neither controlled her head nor babbled. Because we suspected chromosomal abnormalities as a cause of multi-system abnormalities, growth restriction and developmental delay, G-banding analysis was performed at age 7 months. At this point, 46,XY karyotype was unexpectedly revealed. Endocrinological investigation at 7 months showed a normal serum cortisol level (10.9 μg/dL) with a normal plasma ACTH level (47.9 pg/mL). The levels of LH (3.2 U/L), FSH (6.4 U/L) and testosterone (0.89 ng/mL) were interpreted as normal considering her corrected age. Abdominal ultrasonography showed normal-sized adrenal glands. We did not perform an ACTH stimulation test. At age 11 months, she was discharged home from the neonatal intensive care unit. Soon after the discharge, she was re-hospitalized due to pneumonia accompanied with altered consciousness. She was then subjected to mechanical ventilation. At age 14 months, she suffered from gastrointestinal dysmotility accompanied by hematochezia, and died presumably due to hypovolemic shock.

### Histological analysis of the placenta

To unveil the mechanisms of SGA and DSD in MIRAGE syndrome, we performed histological examination of placenta tissues derived from the patient with the p.Phe1017Val variant. We also obtained and studied placenta tissues of a previously published patient with p.Arg459Gln variant [5]. Histological findings were consistent with distal villous hypoplasia, namely sparse, poorly developed distal villous tree with widening of the intervillous space (Fig 4C). Intermediate villi were edematous. Neither chorioamnionitis, fetal thrombotic vasculopathy, placental infarction nor decidual vasculopathy were observed.

## Discussion

The aim of this study was to screen *SAMD9* variants in a patient cohort presenting partial MIRAGE phenotype, in particular those who do not have AI. We choose 46,XY DSD SGA as a target cohort, because co-existence of 46,XY DSD and SGA is occasionally encountered [2]. By genetic screening, we could identify only one *SAMD9* variant carrier among the 49 subjects, indicating that pathogenic *SAMD9* variants are a rare cause of 46,XY DSD SGA without AI. Importantly, the variant-carrying patient was not only affected by DSD and SGA, but also had multiple complications including transfusion-requiring thrombocytopenia, postnatal growth restriction, gastrointestinal problems, and susceptibility to severe infection. Overall, the patient had all MIRAGE features except for adrenal hypoplasia, and it seems natural to recognize her as having MIRAGE syndrome. Although accumulation of clinical experience is needed to draw firm conclusions, we suppose that clinicians should consider *SAMD9* variants in 46,XY DSD SGA patients with multiple comorbidities.

The *SAMD9* variant-carrying patient described in the present study had no apparent symptoms or laboratory findings suggesting AI. Very recently, a total of ten *SAMD9* variant carriers with seemingly no adrenal problems were discovered among pediatric patients with bone marrow failure or myelodysplastic syndrome [13, 14]. These observations, along with our experience, suggest that the adrenal phenotype could be variable in MIRAGE syndrome. There are at least two possible explanations for the variability. First, genetic forms of congenital adrenal hypoplasia, such as *NR0B1* variants [15] and IMAGe syndrome [16] are known to show variable presentation of AI in terms of onset age and disease severity. This rule could be valid for *SAMD9* variants. In general, multi-system disorders can have variable clinical presentations, and thus lack of a single feature does not necessarily preclude a diagnosis. A second possibility is “reversion mutations” that alleviate the deleterious effect of *SAMD9* variants. Inactivation of the disease-causing *SAMD9* allele by somatic acquisition of an *in ci*s second-site inactivating variant have been repeatedly observed in blood cells of MIRAGE syndrome patients [6, 12]. Considering the very high regenerating capacity of the adrenal gland best demonstrated by transplantation experiments among rats [17], a similar rescue mechanism can occur in the adrenal gland as Buonocore *et al*. have already pointed [6]. Even though presentation of AI could be variable in MIRAGE syndrome, we still regard it as an important diagnostic clue, because AI is by far the most uncommon medical condition among the six core features.

**Fig 5:**
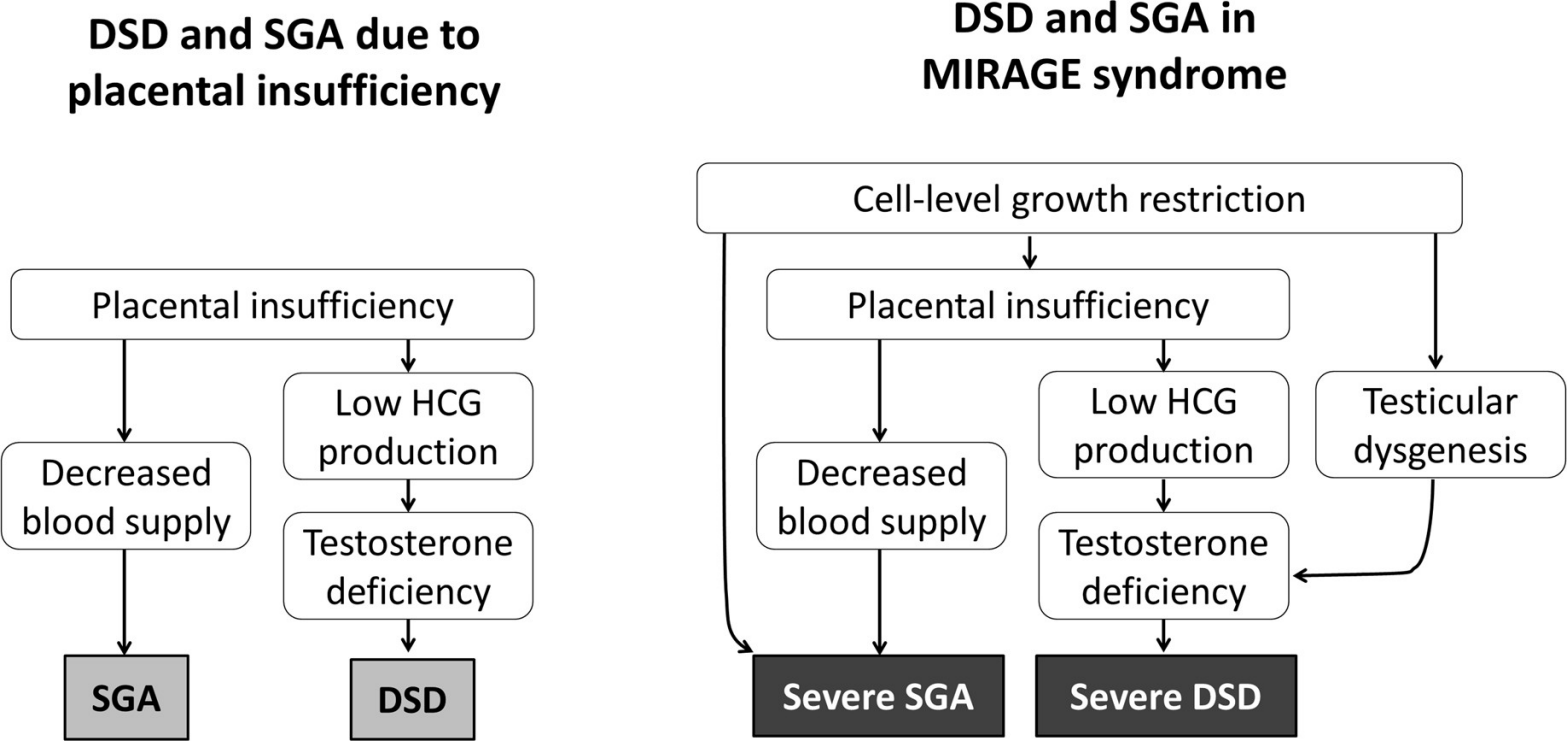
Schematic diagrams showing the mechanism of SGA and DSD in simple placental insufficiency and MIRAGE syndrome. SGA and DSD can be resulted from placental insufficiency (*Left panel*). In MIRAGE syndrome, cell-level growth restriction, which is a direct effect of a *SAMD9* variant, cause additional negative effect on fetal growth and testicular development, giving rise to more severe phenotype than simple placental insufficiency.

We performed the first histological analyses of the placenta tissues derived from MIRAGE syndrome patients, revealing characteristic placental villous maldevelopment. Mutated SAMD9 proteins have potent growth-restricting capacity, and thus they can directly cause systemic growth restriction and testicular hypoplasia. Moreover, *SAMD9* variants also affect placenta, resulting in poor blood supply and suboptimal HCG stimulation. MIRAGE syndrome patients found in the present study and in previously published studies [5, 6, 12, 18] had more severe genital phenotypes than most variant-negative 46,XY DSD SGA patients enrolled in this study. It seems reasonable to hypothesize that coexistence of the two mechanisms (direct effect by a pathogenic variant and indirect effect by placental insufficiency) would be responsible for the severe clinical phenotypes (Fig 5).

In conclusion, we conducted the first genetic screen of *SAMD9* variants in a patient cohort of 46,XY DSD SGA without AI, and showed that MIRAGE syndrome is a rare cause of the condition. AI is a common feature of MIRAGE syndrome, but the absence of AI should not rule out a diagnosis of MIRAGE syndrome. The pathogenesis of SGA and DSD in MIRAGE syndrome seems multifactorial, including intrinsic growth restriction and placental insufficiency both related to pathogenic *SAMD9* variants.
